# slan/M-DC8^+^ cells constitute a distinct subset of dendritic cells in human tonsils

**DOI:** 10.18632/oncotarget.6660

**Published:** 2015-12-18

**Authors:** Alessandra Micheletti, Giulia Finotti, Federica Calzetti, Silvia Lonardi, Elisa Zoratti, Mattia Bugatti, Stefania Stefini, William Vermi, Marco A. Cassatella

**Affiliations:** ^1^ Department of Medicine, Section of General Pathology, University of Verona, Verona, Italy; ^2^ Department of Molecular and Translational Medicine, Section of Pathology, University of Brescia, Brescia, Italy; ^3^ Applied Research on Cancer-Network (ARC-NET), University of Verona, Verona, Italy; ^4^ Unit of Pediatric Otorhinolaryngology, Spedali Civili di Brescia, Brescia, Italy; ^5^ Department of Pathology and Immunology, Washington University School of Medicine, Saint Louis, Missouri, USA

**Keywords:** slan/M-DC8^+^ cells, dendritic cells, monocytes, tonsil, differentiation, Immunology and Microbiology Section, Immune response, Immunity

## Abstract

Human blood dendritic cells (DCs) include three main distinct subsets, namely the CD1c^+^ and CD141^+^ myeloid DCs (mDCs) and the CD303^+^ plasmacytoid DCs (pDCs). More recently, a population of slan/M-DC8^+^ cells, also known as “slanDCs”, has been described in blood and detected even in inflamed secondary lymphoid organs and non-lymphoid tissues. Nevertheless, hallmarks of slan/M-DC8^+^ cells in tissues are poorly defined. Herein, we report a detailed characterization of the phenotype and function of slan/M-DC8^+^ cells present in human tonsils. We found that tonsil slan/M-DC8^+^ cells represent a unique DC cell population, distinct from their circulating counterpart and also from all other tonsil DC and monocyte/macrophage subsets. Phenotypically, slan/M-DC8^+^ cells in tonsils display a CD11c^+^HLA-DR^+^CD14^+^CD11b^dim/neg^CD16^dim/neg^CX3CR1^dim/neg^ marker repertoire, while functionally they exhibit an efficient antigen presentation capacity and a constitutive secretion of TNFα. Notably, such DC phenotype and functions are substantially reproduced by culturing blood slan/M-DC8^+^ cells in tonsil-derived conditioned medium (TDCM), further supporting the hypothesis of a full DC-like differentiation program occurring within the tonsil microenvironment. Taken together, our data suggest that blood slan/M-DC8^+^ cells are immediate precursors of a previously unrecognizedcompetent DC subset in tonsils, and pave the way for further characterization of slan/M-DC8^+^ cells in other tissues.

## INTRODUCTION

Dendritic cells (DCs) represent a heterogeneous population of myeloid cells that are characterized by a very efficient capacity to present antigens to T cells. To date, three types of blood DCs, deriving from the same precursor [1], have been described in humans [2]. Specifically, the plasmacytoid DCs (pDCs), that are specialized in type I interferon production [3], and the conventional myeloid DCs (mDCs), that include the CD1c^+^(BDCA1^+^) DCs and the CD141^+^(BDCA3^+^) DCs, the latter ones being skilled at antigen cross-presentation to CD8^+^ T cells [4]. All these DC populations have been also found in secondary lymphoid organs, including tonsils, spleen and lymph nodes [5-7]. An additional population of blood myeloid cells, that shares a number of phenotypic and functional characteristics with classical mDCs, has been described and called “slanDCs” by Schäkel and colleagues [8]. Accordingly, slanDCs have been identified by the use of a specific monoclonal antibody (M-DC8) recognizing the 6-Sulfo LacNAc (slan) carbohydrate modification of PSGL-1, whose acronym gave thus origin to the “slanDC” terminology [9-10]. However, on a two-dimensional flow cytometry dot plot of CD14 and CD16 expression in peripheral blood mononuclear cells (PBMCs), slan/M-DC8^+^ cells in part overlap with CD14^dim^CD16^+^ monocytes [10-11], suggesting that they might actually represent a subset of non-classical monocytes [12-13]. Functionally, blood slan/M-DC8^+^ cells have been described as potent pro-inflammatory cells based on their capacity to produce large amount of tumor necrosis factor alpha (TNFα) and IL-12p70 upon stimulation with toll-like receptor (TLR) ligands [10, 14]. Blood slan/M-DC8^+^ cells also promote proliferation, cytotoxicity and interferon-gamma (IFNg) production by natural killer (NK) cells [8, 15-16], and induce strong antigen-specific T-cell responses [9]. Furthermore, it is well established that slan/M-DC8^+^ cells locate in lymphoid and peripheral tissues, especially under inflammatory conditions. slan/M-DC8^+^ cells, in fact, have been identified in mucosal associated lymphoid tissue (such as tonsils [17],[11] and intestine Peyer's patches [17]), in skin of inflammatory diseases including lupus erythematosus [18] and psoriasis [14], in the colonic mucosa of Crohn disease patients [16-17], as well as in carcinoma-draining lymph nodes [11]. However, even though blood slan/M-DC8^+^ cell function and phenotype have been exhaustively delineated, an extensive comparison between blood and tissue slan/M-DC8^+^ cells, as well as between tissue slan/M-DC8^+^ cells and other tissue DC/macrophage populations, has never been performed.

In this study, we have performed a detailed characterization of slan/M-DC8^+^ cells in tonsils, in turn demonstrating that they represent a unique DC population, clearly different from any other tonsil DC or monocyte/macrophage population described to date [19]. Moreover, our data suggest that blood slan/M-DC8^+^ cells contribute to replenish such slan/M-DC8^+^ DC pool in tonsils, thus uncovering new information on plasticity by blood slan/M-DC8^+^ cells and their ultimate commitment within tissue microenvironments.

## RESULTS

### slan/M-DC8^+^ cells as a unique DC population in human tonsils

**Figure 1:**
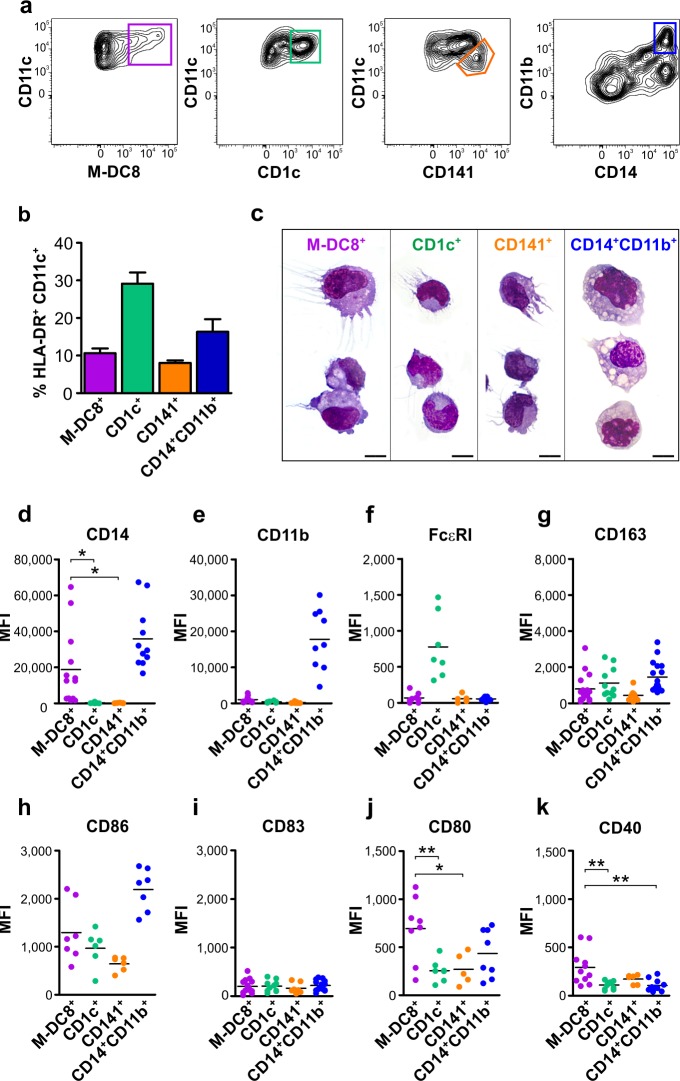
Phenotypic characterization of slan/M-DC8^+^ DCs and other myeloid populations in human tonsils **a.** Contour plots illustrate how slan/M-DC8^+^ DCs, as well as CD1c^+^ DCs, CD141^+^ DCs and CD14^+^CD11b^+^ monocytes/macrophages, were identified within tonsil cell suspensions by flow cytometry (a more complete and detailed gating strategy is reported in Supplementary Figure S1). **b.** Graph shows the percentages of tonsil slan/M-DC8^+^ DCs, CD1c^+^ DCs, CD141^+^ DCs and CD14^+^CD11b^+^ monocytes/macrophages among all HLA-DR^+^CD11c^+^ myeloid cells (*n* = 15-20). **c.** Morphology of sorted slan/M-DC8^+^ DCs, CD1c^+^ DCs, CD141^+^ DCs and CD14^+^CD11b^+^ monocytes/macrophages on cytospins stained by May-Grunwald Giemsa (scale bar = 20 μm). **d.**-**k.** Graphs show the expression levels of each indicated marker in tonsil slan/M-DC8^+^ DCs, CD1c^+^ DCs, CD141^+^ DCs and CD14^+^CD11b^+^ monocytes/macrophages, as measured by flow cytometry. Values indicate the mean fluorescence intensity (MFI) for each sample. *P < 0.05; **P < 0.01, by one-way ANOVA test.

**Figure 2:**
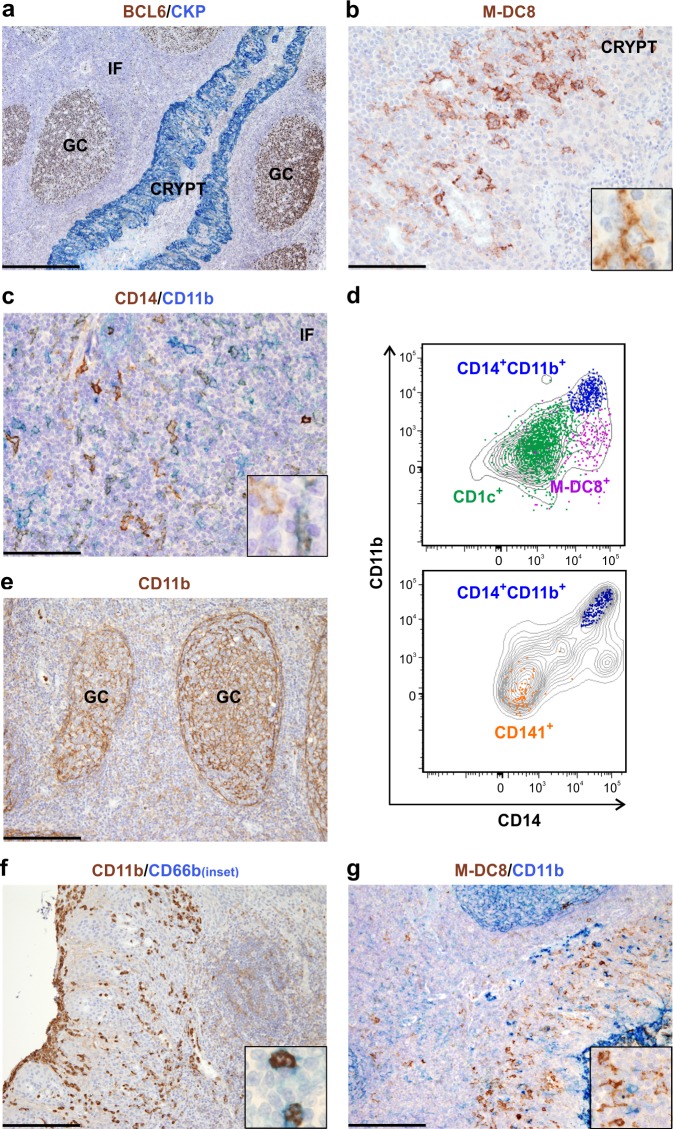
slan/M-DC8^+^ DCs and CD14^+^CD11b^+^ monocytes/macrophages are distinct cell populations in human tonsils **a.**-**c.**; **e.**-**g.** Sections are from tonsil samples and stained as indicated by labels. **a**. Pan-cytokeratin (CKP) and BCL6 identify different compartments including follicles with BCL6^+^ germinal centre (GC) B-cells, CKP^+^ epithelial crypts and the interfollicular area (IF) between two or more follicles. **b.** High power view of a tonsil crypt area showing slan/M-DC8^+^ DCs intermingled with epithelial cells. Inset shows a higher magnification of slan/M-DC8^+^ DC morphology. **c.** High power view of an interfollicular area showing a CD14/CD11b double staining. Inset shows a higher magnification of a CD14^+^ cell as well as a CD14^+^CD11b^+^ cell. **e.**, **f.** CD11b stains both follicular DCs in germinal centers (e), and CD66b^+^ neutrophils in the tonsil epithelium (f); inset in panel f shows a high power view of CD11b^+^CD66b^+^ neutrophils. (**g**. and inset) Tonsil slan/M-DC8^+^ DCs are instead completely negative for CD11b. Sections are counterstained with Meyer's haematoxylin. Original magnifications: 40X (panel a, scale bar 500 μm); 100X (panels e-g, scale bar 200 μm); 200X (panels b,c, scale bar 100 μm); 600X (insets). **d.** Overlay plots displaying the CD11b and CD14 levels in tonsil slan/M-DC8^+^ DCs, CD1c^+^ DCs, CD141^+^ DCs and CD14^+^CD11b^+^ monocytes/macrophages, as measured by flow cytometry. Single cell populations were first identified by specific markers (as depicted in Figure 1a) and then overlaid on the contour plots of total CD11c^+^HLA-DR^+^ cells. A representative experiment, out of at least 4 performed with similar results, is shown.

To better characterize the frequency, phenotype, differentiation state and function of slan/M-DC8^+^ cells in tissues, we initially analyzed, by flow cytometry, single cell suspensions from a large set of human tonsils. All tonsil samples were obtained from children affected by recurrent, chronic tonsillitis. Using the gating strategy illustrated in Supplementary Figure S1, among HLA-DR^+^CD11c^+^ myeloid cells we could identify two DC populations, namely the CD1c^+^(BDCA-1^+^) DCs and the CD141^+^(BDCA-3^+^) DCs (Figure 1a), as previously reported by others [19], and a CD14^+^CD11b^+^ monocyte/macrophage population. In addition, we could also identify the slan/M-DC8^+^ cells (Figure 1a). We calculated that the slan/M-DC8^+^ cells account for about 0.1 % of the total CD45^+^ leukocytes (data not shown), and about 10 % of the total HLA-DR^+^CD11c^+^ myeloid cells in tonsils (Figure 1b). In such regard, slan/M-DC8^+^ cell frequency was found similar to that of CD141^+^ DCs (8.1 ± 3.1 %; *n* = 22), but consistently lower than those of CD1c^+^ DCs (29.2 ± 13.5 %; *n* = 21) or CD14^+^CD11b^+^ monocytes/macrophages (16.3 ± 13 %; *n* = 15) (Figure 1b). As assessed by cytospin preparations of sorted cells, tonsil slan/M-DC8^+^ cells displayed a typical DC shape, similar to CD1c^+^ and CD141^+^ DCs, yet showing a larger size (Figure 1c). Conversely, CD14^+^CD11b^+^ monocytes/macrophages consist of a heterogeneous population that includes large cells with typical macrophage morphology, containing phagocytic vacuoles admixed to smaller cells with round morphology and similar to monocytes (Figure 1c). Among the different tonsil compartments identified by the BCL6/CKP staining (Figure 2a), slan/M-DC8^+^ cells were found mainly located in the crypts (Figure 2b), as previously reported [11], while CD14^+^CD11b^+^ monocytes/macrophages were predominant in the inter-follicular (IF) area (Figure 2c).

By characterizing their phenotype by flow cytometry, we observed that, despite donor variability, and in contrast to their blood counterpart, tonsil slan/M-DC8^+^ cells did express CD14, a feature shared with monocytes/macrophages (Figures 1d and 2d). By contrast, CD11b was found neither in slan/M-DC8^+^ cells, nor in other DCs (Figures 1e and 2d). Moreover, by IHC staining of tonsil sections, the anti-CD11b antibody strongly stained follicular DCs (Figure 2e), neutrophils (Figure 2f) and a population of small mononuclear cells (likely monocytes, Figure 2g), but not slan/M-DC8^+^ cells (Figure 2g). A weak CD11b reactivity was also observed in larger CD14^+^ mononuclear cell in the IF area (Figure 2c), therefore accounting for the CD11b^+^CD14^+^ population detectable by flow cytometry (Figure 1a).

The possibility that tonsil slan/M-DC8^+^ cells might overlap with a recently identified population of CD14^+^FcεRI^+^ present in human inflammatory fluids, and able to induce Th17 differentiation [20], was also excluded since tonsil slan/M-DC8^+^ cells do not express FcεRI (Figure 1f). Interestingly, we could observe that FcεRI is, however, expressed by tonsil CD1c^+^ DCs (Figure 1f), which are instead CD14-negative (Figures 1d and 2d). By flow cytometry, we found that CD163, previously reported as a marker for axillary lymph node CD14^+^ cells [7], was variably expressed by all cell populations under investigation (Figure 1g). Finally, analysis of costimulatory molecule expression revealed that, while CD86 was expressed in slan/M-DC8^+^ cells, mDCs and CD11b^+^CD14^+^ monocytes/macrophages (Figure 1h), CD83 was regularly absent in all these cell populations (Figure 1i). Notably, both CD40 and CD80 were expressed at the highest levels in tonsil slan/M-DC8^+^ cells (Figure 1j, 1k). Finally, we found that tonsil slan/M-DC8^+^ cells do not express CD206 and CD209 (data not shown). Altogether, these data qualify tonsil slan/M-DC8^+^ cells as a distinct DC population. Data also suggest that, by flow cytometry, CD11b could be a much more useful marker to distinguish tonsil CD11b^dim/neg^ DC subsets from tonsil CD11b^bright^ monocytes/macrophages than the commonly used CD14 or CD163.

### Blood slan/M-DC8^+^ cells incubated in tonsil-derived conditioned medium (TDCM) acquire the phenotype of tonsil slan/M-DC8^+^ DCs

**Figure 3:**
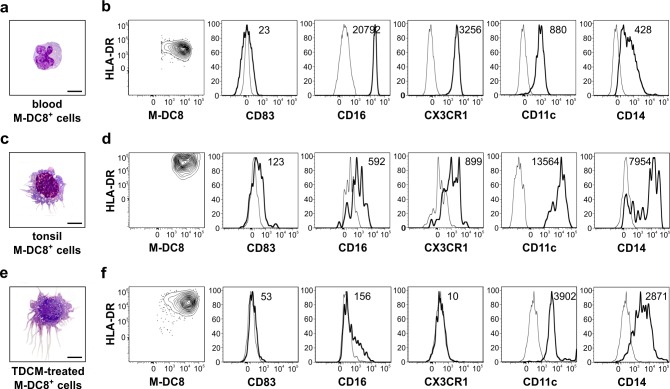
Blood slan/M-DC8^+^ cells incubated in tonsil derived-conditioned medium (TDCM) acquire the morphology and phenotype of tonsil slan/M-DC8^+^ DCs Blood slan/M-DC8^+^ cells were cultured for 5 d in TDCM and then compared to tonsil slan/M-DC8^+^ DCs in terms of morphology and phenotype. Morphology of, respectively, blood **a.**, tonsil-sorted **c.**, and TDCM-conditioned **e.**, slan/M-DC8^+^ cells on cytospins stained by May-Grunwald Giemsa (scale bar = 20 μm) is shown. **b.**, **d.**, **f.** Contour plots and histograms illustrating the expression of each indicated marker (thick black line) versus related isotype control (thin black line) in blood **b.**, tonsil-sorted **d.** and TDCM-conditioned **f.** slan/M-DC8^+^ cells. MFI value for each marker is also reported in corresponding histogram. A representative experiment out of 4 performed with similar results is shown.

A comparative analysis between blood versus tonsil slan/M-DC8^+^ cells revealed substantial differences in morphology and phenotype. In fact, blood slan/M-DC8^+^ cells are round with irregularly shaped nucleus (Figure 3a), while slan/M-DC8^+^ DCs purified from tonsils are larger cells with large round nuclei and acquire dendrites (Figures 1c and 3c). Phenotypically, blood and tonsil slan/M-DC8^+^ cells are CD83-negative and maintain equivalent levels of M-DC8 (Figure 3b, 3d). By contrast, tonsil slan/M-DC8^+^ DCs express lower levels of both CD16 and CX3CR1, but higher levels of HLA-DR, CD11c and CD14 than blood slan/M-DC8^+^ cells (Figure 3b, 3d), thus suggesting that the latter cells modify their phenotype once recruited into tonsils.

Concomitantly with the analysis of *ex vivo* isolated tonsil slan/M-DC8^+^ DCs, we set up an *in vitro* model aimed at inducing a tonsil-like phenotype in slan/M-DC8^+^ cells purified from the blood of healthy donors. Specifically, we generated various TDCMs and used them as a culture medium for blood slan/M-DC8^+^ cells. As shown in Figure 3e, blood slan/M-DC8^+^ cells conditioned by TDCM for 5 days become morphologically very similar to slan/M-DC8^+^ DCs directly purified from tonsils (Figure 3c). We also observed that TDCM-conditioned slan/M-DC8^+^ cells down-modulated CD16 and CX3CR1, while they up-regulated HLA-DR, CD11c and CD14 (Figure 3f), thus mirroring the phenotype of freshly purified tonsil slan/M-DC8^+^ DCs (Figure 3d). Accordingly, CD83 remained negative also in TDCM-conditioned slan/M-DC8^+^ cells (Figure 3f). Taken together, these experiments demonstrate that TDCM substantially induces a tonsil-like phenotype in blood slan/M-DC8^+^ cells, thus supporting the hypothesis of a “differentiation program” that peripheral slan/M-DC8^+^ cells undertake upon their arrival in tonsils.

### Blood slan/M-DC8^+^ cells exhibit a remarkable plasticity

**Figure 4:**
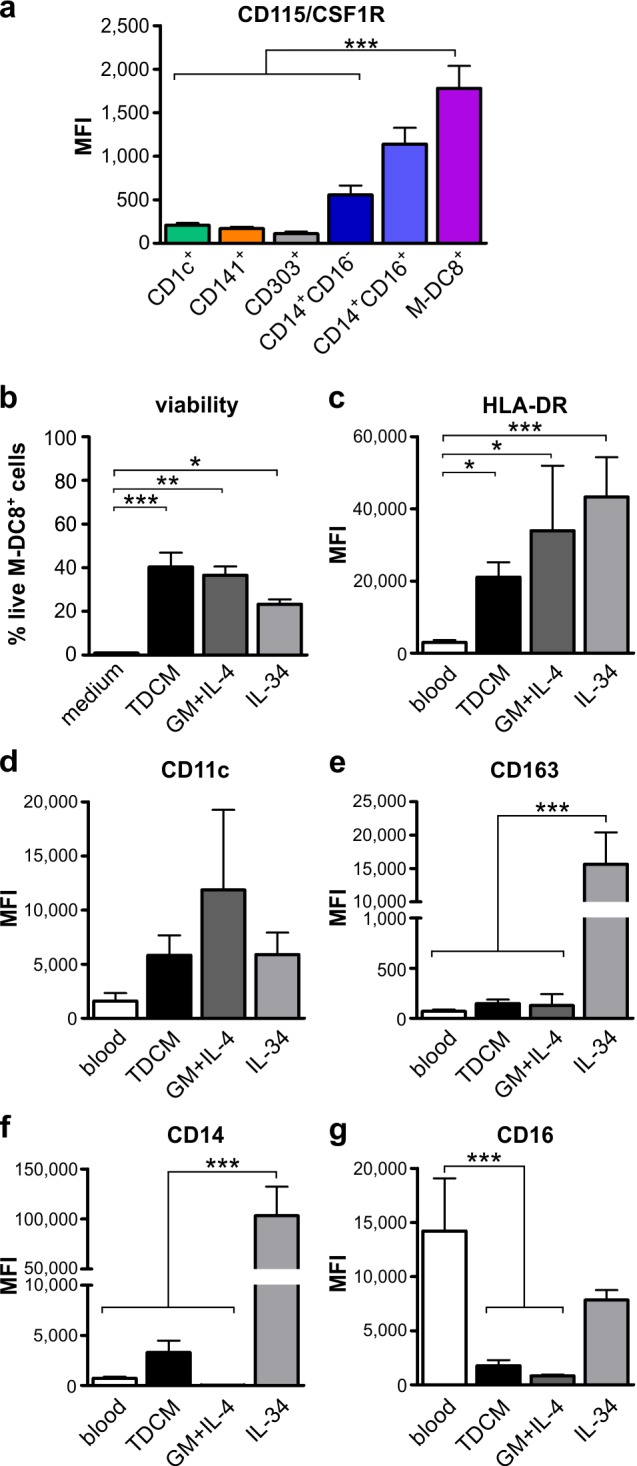
Blood slan/M-DC8^+^ cells display the capacity to polarize toward either a “DC-like” or a “macrophage-like” phenotype **a.** Expression levels of CD115/CSF1R in blood CD1c^+^, CD141^+^, CD303^+^, CD14^+^CD16^−^, CD14^+^CD16^+^ and slan/M-DC8^+^ cells within freshly isolated peripheral PBMCs (*n* = 4). **b.**-**g.** Blood slan/M-DC8^+^ cells were cultured for 5 days in: medium alone (only in panel b), tonsil-derived conditioned medium (TDCM), 50 ng ml^−1^ GM-CSF plus 20 ng ml^−1^ IL-4, or 100 ng ml^−1^ IL-34. **b.** Graph shows the percentage of live slan/M-DC8^+^ cells after a 5 d-incubation under each stimulatory condition (*n* = 8-10). Cell viability was established by flow cytometry, using Vybrant^®^ DyeCycle™ Violet Stain. Live cells were gated (e.g., Vybrant negative slan/M-DC8^+^ cells) and surface marker expression then analyzed. **c.**-**g.** Graphs show the levels of expression of HLA-DR (c), CD11c (d), CD163 (e), CD14 (f) and CD16 (g) in 5 d-treated slan/M-DC8^+^ cells and freshly purified blood slan/M-DC8^+^ cells (*n* = 8-15). *P < 0.05; **P < 0.01; ***P < 0.001, by one-way ANOVA test.

In subsequent experiments, we compared the phenotype of TDCM-conditioned slan/M-DC8^+^ cells with the phenotypes acquired by blood slan/M-DC8^+^ cells incubated for 5 days in the presence of either GM-CSF plus IL-4, which is known to generate competent DCs from circulating slan/M-DC8^+^ cells [21], or IL-34, which induces a macrophage differentiation from classical CD14^+^ monocytes [22]. Notably, blood slan/M-DC8^+^ cells express the highest levels of CD115/CSF1R (e.g., the receptor shared by both M-CSF and IL-34) as compared to the other blood DC and monocyte subsets (Figure 4a). First of all, we found that, unlike control medium, all stimulatory conditions maintained the survival of slan/M-DC8^+^ cells at variable levels (Figure 4b). Then, we observed that culturing slan/M-DC8^+^ cells with GM-CSF plus IL-4, IL-34 or TDCM, significantly up-regulated the expression of HLA-DR, in line with an *in vitro*-induced differentiation process (Figure 4c). A similar trend was also observed for the expression of CD11c (Figure 4d), even though its modulation did not reach statistical significance. Interestingly, surface CD163, CD14 and CD16, which are typically co-expressed by macrophages [22-23], were either upregulated (CD163 and CD14) or maintained (CD16) in IL-34-treated slan/M-DC8^+^ cells (Figure 4e-4g). Conversely, the same three markers were almost negative when slan/M-DC8^+^ cells where cultured in GM-CSF plus IL-4 (Figure 4e-4g), in line with their DC-like differentiation [21, 24]. In such regard, TDCM-conditioned slan/M-DC8^+^ cells, as GM-CSF plus IL-4-conditioned slan/M-DC8^+^ cells, did express either CD163 or CD16 at minimal levels (Figure 4e, 4g). Finally, TDCM-conditioned slan/M-DC8^+^ cells were found to express moderate amounts of CD14 (Figure 4f), yet at significantly higher levels than their blood counterpart (*P* < 0.001 by two-tailed unpaired t test), consistent with the CD14 detection in tonsil slan/M-DC8^+^ DCs (Figure 1d). Our data demonstrate that TDCM-conditioned slan/M-DC8^+^ cells display a DC-like antigen expression profile that is more similar to that acquired by GM-CSF plus IL-4-conditioned slan/M-DC8^+^ cells than to the macrophage-like one induced by IL-34. Interestingly, we found that GM-SCF, but not IL-4, was detectable in all TDMCs used for our *in vitro* differentiation (ranging from 200 pg ml^−1^ to 2800 pg ml^−1^). Taken together, data also uncover that blood slan/M-DC8^+^ cells exhibit a remarkable plasticity and differentiate into either DCs or macrophages, depending on the type of differentiation factors they are exposed to.

### Tonsil slan/M-DC8^+^ DCs efficiently present antigens to T cells

**Figure 5:**
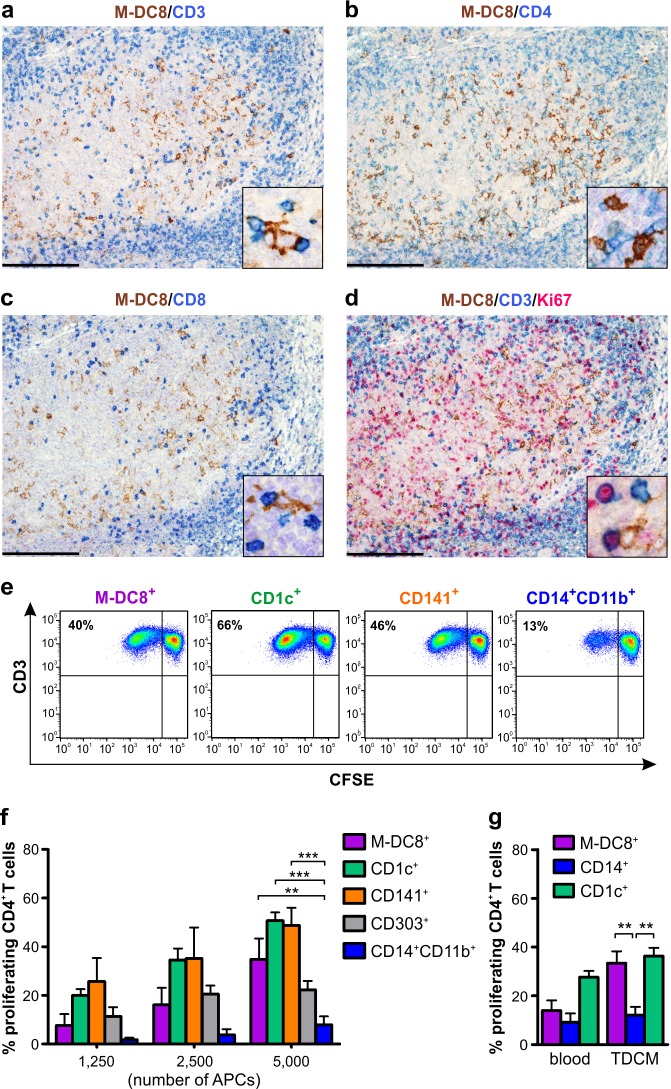
slan/M-DC8^+^ DCs interact with T cells in tonsils and display a remarkable antigen presentation capacity **a.**-**d.** Sections are from human tonsils stained as indicated by labels. Double staining shows that a fraction of slan/M-DC8^+^ DCs interact with CD3^+^ T cells in the crypt (a), which are either CD4^+^ (as dominant population, b) and CD8^+^ (c). **d**. Triple staining shows that the T cell population interacting with slan/M-DC8^+^ DCs includes a fraction of CD3^+^Ki67^+^ proliferating T lymphocytes. Cell interactions are illustrated by high power view insets in panels a-d. Sections are counterstained with Meyer's haematoxylin. Original magnifications: 100X (panels a-d, scale bar 200 μm); 600X (insets in a-d). **e.**, **f.** Sorted tonsil slan/M-DC8^+^ DCs, CD1c^+^ DCs, CD141^+^ DCs, CD14^+^CD11b^+^ monocytes/macrophages and CD303^+^ pDCs were co-cultured with CFSE-labeled allogeneic CD4^+^ T cells for 7 days. T cell proliferation was then determined by the CFSE dilution method. **e.** Representative experiment displaying T cell proliferation by the CSFE assay, in which 5×10^4^ T cells were cultured with 5×10^3^ cells of sorted tonsil slan/M-DC8^+^ DCs, CD1c^+^ DCs, CD141^+^ DCs, CD14^+^CD11b^+^ monocytes/macrophages in a final volume of 200 μL. **f.** Graph shows the % of T cell proliferation induced by an increasing number of each tonsil cell population, as indicated (*n* = 3-7). **P < 0.01; ***P < 0.001, by two-way ANOVA test. **g.** 5×10^3^ freshly isolated (blood) or 5-d TDCM-conditioned slan/M-DC8^+^ cells, CD14^+^ monocytes or CD1c^+^ DCs from the same healthy donors were co-cultured with 5×10^4^ CFSE-labeled autologous CD4^+^ T cells in the presence of TT antigen in a final volume of 200 μL, for 7 days. Graph shows the % of T cell proliferation induced by each cell population (*n* = 3-7). **P < 0.01, by two-way ANOVA test.

Extending previous observations [11], double stains for M-DC8 and CD3, CD4 or CD8 (Figure 5a-5c) confirmed that, in human tonsils, slan/M-DC8^+^ DCs interact with T cells. In addition, some CD3^+^ T cells contacting slan/M-DC8^+^ DCs also co-stain for the proliferating marker Ki67 (Figure 5d). Based on these findings, we then analyzed the Ag presentation capacity by slan/M-DC8^+^ DCs isolated from tonsils. We thus sorted CD11c^+^slan/M-DC8^+^ DCs along with all other DC/macrophage populations and then cultured each cell type with allogeneic CD4^+^ T lymphocytes to measure their proliferation after 7 days (Figure 5e, showing a representative experiment). We observed that, at least at their highest concentrations, tonsil slan/M-DC8^+^ DCs displayed, similarly to CD1c^+^ or CD141^+^ DCs, an Ag presentation capacity significantly higher than tonsil CD14^+^CD11b^+^ monocytes/macrophages. The latter cells, indeed, were reproducibly found to be very poor stimulatory APCs for T cells (Figure 5e, 5f).

Subsequently, we analyzed the capacity of TDCM-conditioned slan/M-DC8^+^ cells to perform Ag presentation under autologous settings. We thus co-cultured blood and TDCM-conditioned slan/M-DC8^+^ cells with autologous CD4^+^ T cells for 7 d in the presence of Tetanus Toxoid (TT). We observed that TDCM-conditioned slan/M-DC8^+^ cells induced a CD4^+^ T cell proliferation at higher extent than freshly isolated, autologous blood slan/M-DC8^+^ cells, while peripheral CD14^+^ monocytes (either freshly isolated or conditioned with TDCM) resulted to be poor APCs (Figure 5g). Donor-matched blood CD1c^+^ DCs performed the strongest Ag presentation capacity without the necessity to differentiate. Indeed, freshly isolated as well as TDCM-conditioned CD1c^+^ DCs promoted an equivalent T cell proliferation (Figure 5g). Of note, the Ag presentation capacity by TDCM-conditioned slan/M-DC8^+^ cells (Figure 5g) and freshly purified tonsil slan/M-DC8^+^ DCs cultured at the same concentration (e.g., 5,000 APCs) were similar (Figure 5f). Taken together, data support the notion that tonsil slan/M-DC8^+^ DCs represent an additional bona fide DC subset present in tonsils. Data also demonstrate that TDCM could be used as a valid *in vitro* model to induce, starting from blood slan/M-DC8^+^ cells, not only the phenotype but also the APC function of tonsil slan/M-DC8^+^ DCs.

### Tonsil slan/M-DC8^+^ DCs constitutively secrete TNFα but not IL-12p70

**Figure 6:**
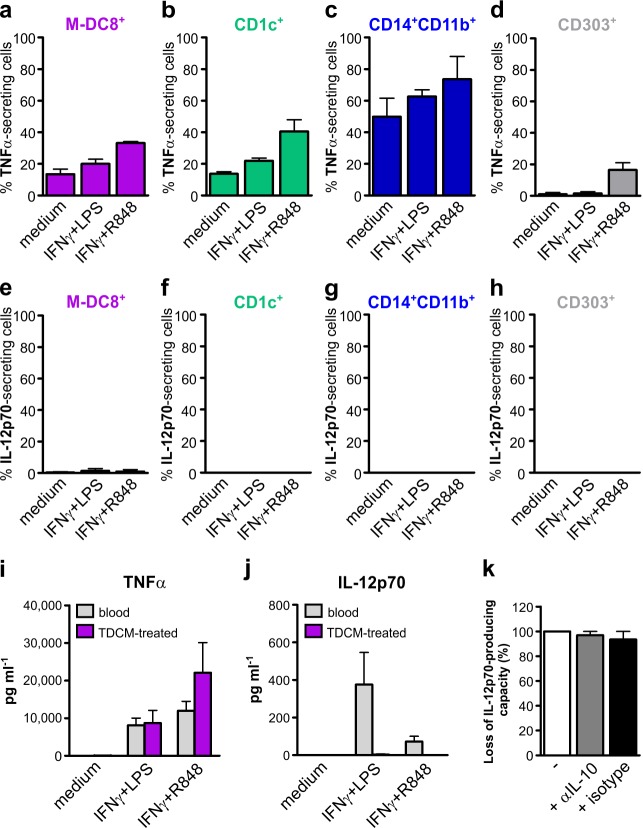
Tonsil slan/M-DC8^+^ DCs, CD1c^+^ DCs, CD14^+^CD11b^+^ monocytes/macrophages and CD303^+^ pDCs produce TNFα but not IL-12p70 **a.**-**h.** Tonsil cell suspensions were incubated with or without 100 U ml^−1^ IFNγ plus either 100 ng ml^−1^ LPS or 5 μM R848, either for 4 h (to detect TNFα secretion, a-d), or for 12 h, after a 6 h pre-incubation (to detect IL-12p70 secretion, e-h). Graphs show TNFα-secreting slan/M-DC8^+^ DCs **a.**, CD1c^+^ DCs **b.**, CD14^+^CD11b^+^ monocytes/macrophages **c.** and CD303^+^ pDCs **d.**, or IL-12p70-secreting slan/M-DC8^+^ DCs **e.**, CD1c^+^ DCs **f.**, CD14^+^CD11b^+^ monocytes/macrophages **g.** and CD303^+^ pDCs **h.**. The graphs show the mean of cytokine secreting cells (as percentage of each cell population) calculated from 4 experiments. **i.**, **j.** 2.5×10^4^ 100 μl^−1^ blood (gray bars), or 5-d TDCM-conditioned (purple bars), slan/M-DC8^+^ cells were incubated for 24 h with or without 100 U ml^−1^ IFNγ plus either 100 ng ml^−1^ LPS or 5 μM R848 to measure the levels of TNFα (i) and IL-12p70 (j) in cell free supernatants by ELISA (*n* = 5-7). **k.** Blood slan/M-DC8^+^ cells were either immediately stimulated with 100 U ml^−1^ IFNγ plus 100 ng ml^−1^ LPS for 24 h, or conditioned in TDCM, in the presence or absence of 10 μg ml^−1^ anti-IL-10 or IgG2a isotype control mAbs. After 5 d of incubation, TDCM-conditioned slan/M-DC8^+^ cells were stimulated with IFNγ plus LPS for 24 h. IL-12p70 was then measured in cell-free supernatants by ELISA. Graph show the loss of IL-12p70-producing capacity (in %) by TDCM-conditioned slan/M-DC8^+^ cells after IFNγ plus LPS stimulation (in the absence or the presence of neutralizing mAbs), as compared to blood slan/M-DC8^+^ cells incubated with IFNγ plus LPS.

Finally, we analyzed the capacity of tonsil slan/M-DC8^+^ DCs to produce TNFα and IL-12p70. CD1c^+^ DCs, CD14^+^CD11b^+^ monocytes/macrophages and CD303^+^ pDCs were also tested for comparison purposes. Initially, we took advantage of a cytokine secretion assay [11], since it allows the direct analysis of cytokine secretion at a single-cell level within a heterogeneous cell population. We found that tonsil slan/M-DC8^+^ DCs constitutively secrete TNFα (Figure 6a and Supplementary Figure S2, this latter showing one representative experiment), unlike blood slan/M-DC8^+^ cells [11]. A constitutive TNFα production was also observed in CD1c^+^ DCs (Figure 6b) and, at higher levels, in CD14^+^CD11b^+^ monocytes/macrophages (Figure 6c), but not in CD303^+^ pDCs (Figure 6d). Stimulation with TLR agonists in combination with IFNγ slightly increased TNFα secretion in slan/M-DC8^+^ DCs, CD1c^+^ DCs and CD14^+^CD11b^+^ monocytes/macrophages (Figure 6a-6c). An induction of TNFα production in CD303^+^ pDCs was instead observed only after R848 stimulation (Figure 6d), consistent with the absence of TLR4 expression by these cells. By contrast, no IL-12p70 secretion could be detected either by tonsil slan/M-DC8^+^ DCs cells, or by the other cell populations, under any experimental condition used (Figure 6e-6h).

Such an *ex vivo* analysis on tonsil slan/M-DC8^+^ DCs was further supported by *in vitro* data using the TDCM-differentiation model. In fact, while blood slan/M-DC8^+^ cells incubated for 24 h with IFNγ plus either LPS or R848 produced both TNFα and IL-12p70 (by ELISA) (Figure 6i, 6j; grey bars), TDCM-conditioned slan/M-DC8^+^ cells retained the capacity to produce only TNFα but not IL-12p70 (Figure 6i, 6j; purple bars).

Given the ability of IL-10 in inhibiting the production of IL-12p70 by monocyte-derived DCs [25-26], we then analyzed whether IL-10 was contained in TDCMs, finding remarkable levels of it (244 ± 179 pg ml^−1^; *n* = 9) in all TDCMs. To clarify whether TDCM-derived IL-10 might be responsible for the loss of IL-12p70 production capacity by activated slan/M-DC8^+^ cells, we therefore added an anti-IL-10 neutralizing antibody to blood slan/M-DC8^+^ cells incubated with TDCM. Then, after 5 d of differentiation, we re-stimulated the cells with IFNγ plus LPS, in the presence of anti-IL-10 neutralizing antibody or its related isotype control, for additional 24 h. As shown in Figure 6k, the inability to produce IL-12p70 by TDCM-conditioned slan/M-DC8^+^ cells under conditions in which IL-10 is neutralized remained unchanged. In control experiments, the same antibody completely restored the IL12p70 production abrogated by exogenous IL-10 (data not shown).

## DISCUSSION

In this study, we have performed an extensive phenotypic and functional characterization of slan/M-DC8^+^ cells in human tonsils, which ultimately proves that these cells represent a unique CD11c^+^HLA-DR^+^CD14^+^CD11b^dim/neg^CD16^dim/neg^CX3CR1^dim/neg^ population of DCs, different from other classical CD1c^+^ and CD141^+^ mDCs or CD14^+^CD11b^+^ monocytes/macrophages. Our data also demonstrate that tonsil slan/M-DC8^+^ DCs differ from their blood counterparts, characterized by a CD11c^+^HLA-DR^+^CD14^dim^CD11b^dim^CD16^bright^CX3CR1^bright^ phenotype, suggesting that blood slan/M-DC8^+^ cells undergo a DC differentiation process once migrated into tonsils. Functionally, tonsil slan/M-DC8^+^ DCs proved to be competent in antigen presentation and to constitutively produce TNFα. Moreover, blood slan/M-DC8^+^ cells incubated with TDCM for 5 days were found to acquire a tonsil-like slan/M-DC8^+^ DC phenotype and function, suggesting the involvement of soluble factors produced by the tonsil environment for such a differentiation process.

slan/M-DC8^+^ cells are usually CD14^dim^ in different compartments (e.g., blood or skin [9, 27]). In this study, an unexpected observation that we uncovered is that, unlike their blood counterpart, tonsil slan/M-DC8^+^ DCs express CD14 at variable but significant levels. This finding is particularly interesting since tissue CD14^+^ myeloid cells are conventionally limited to macrophages (e.g., in human skin [28], tonsils [19], lymph nodes [7], intestine [29] and spleen [30]). Nevertheless, a subset of CD1c^+^FcεRI^+^ inflammatory DCs has been recently reported to express CD14 [20]. Another study also reported the existence of CD14^+^CD163^dim^M-DC8^+^ cells in intestinal lamina propria, displaying features of both macrophages and DCs [31]. Interestingly, this population share, at least in part, the DC phenotype of tonsil slan/M-DC8^+^ DCs herein described. Moreover, a very recent study shows that CD172a^+^ slanDCs in Crohn's disease tissues express CD14 [32]. Taken together, all these findings indicate that the expression of CD14 is not specific for tissue macrophages since it can be also shared by some DC subsets in tissues. By contrast, CD11b was found highly expressed in tonsil CD14^+^ cells but not in slan/M-DC8^+^ DCs or in all other DC populations, suggesting that, at least in tonsils, surface CD11b might better discriminate between DCs and monocytes/macrophages.

In this study, tonsil DCs, including slan/M-DC8^+^ DCs, were found negative for CD83, confirming a previous observation [19]. However, tonsil slan/M-DC8^+^ DCs do express other costimulatory molecules, such as CD40, CD80 and CD86. Moreover, tonsil slan/M-DC8^+^ DCs were found to display a proficient Ag presentation capacity, significantly higher than tonsil CD14^+^CD11b^+^ monocytes/macrophages and similar to other DCs. Thus, despite tonsil M-DC8^+^ cells have been already defined as DCs simply based on their morphology and localization [17], herein we provide the first direct demonstration of their remarkable antigen presentation capacity. Furthermore, our findings are consistent with previous *in vitro* data demonstrating a superior Ag presentation capacity by blood slan/M-DC8^+^ cells than CD14^+^ monocytes [8], as well as a stronger priming activity for naïve T cells by GM-CSF plus IL-4-treated slan/M-DC8^+^ cells than GM-CSF plus IL-4-treated CD14^+^M-DC8^−^ cells [21].

As mentioned, we also show that blood and tonsil slan/M-DC8^+^ cells display a substantially different phenotype. We believe that this is strictly coupled with the slan/M-DC8^+^ cell migration into tonsils and terminal differentiation into DCs. This is also in accordance with the previous demonstration that *in vitro* cultured blood slan/M-DC8^+^ cells, once detaching from erythrocytes (a process mimicking the exit from the vessels), rapidly acquire several characteristics of DCs [8, 10]. Moreover, it has been already reported that CD16^+^ monocytes (which include slan/M-DC8^+^ cells), but not CD14^+^CD16^−^ monocytes, preferentially become DCs in a model of reverse transmigration through endothelial cells [33]. Notably, we also found that tonsil slan/M-DC8^+^ DCs dramatically down-regulate their CD16 expression, which was speculated to represent a step required to differentiate into DCs [21]. Finally, tonsil slan/M-DC8^+^ DCs also down-modulate CX3CR1 expression, a phenomenon that might be caused by its internalization after binding with CX3CL1/Fractalkine, its ligand, which is highly expressed in the crypts of inflamed tonsils [34], where slan/M-DC8^+^ DCs frequently localize [11, 17].

Previous studies have highlighted the proinflammatory nature of circulating slan/M-DC8^+^ cells, for their capacity to produce high levels of TNFα and, particularly, IL-12p70 [9-10], in response to TLR ligands [14]. Immunofluorescence staining of skin lesions from cutaneous lupus erythematosus and psoriasis patients has confirmed that slan/M-DC8^+^ cells are TNFα-positive also in tissues [14, 18]. TNFα expression in colonic mucosa-associated slan/M-DC8^+^ cells of Chron's disease patients has also been reported [32]. Herein, we show a constitutive secretion of TNFα by a fraction of slan/M-DC8^+^ DCs within tonsil cell suspensions, which was also observed to occur in the case of CD1c^+^ DCs and CD14^+^CD11b^+^ monocytes/macrophages, but not CD303^+^ pDCs. Unexpectedly, by using a number of assays, we could not detect any IL-12p70 production either by tonsil slan/M-DC8^+^ DCs or by all other tonsil cell populations under investigations, even after their stimulation with LPS or R848 in the presence of IFNγ. The reasons for such inability to produce IL-12p70 are still unclear and need to be clarified at molecular level. We hypothesize that a general desensitization towards bacterial stimuli [35] might occur in inflamed tonsils continuously exposed to bacteria and their products. This might also explain the concomitant poor responsiveness to LPS/R848 plus IFNγ by *ex vivo* tonsil slan/M-DC8^+^ DCs in terms of TNFα production. Moreover, we explored the possibility that IL-10, readily detectable in our tonsil-conditioned medium, might play a role in determining an inability to produce IL-12p70 by tonsil slan/M-DC8^+^ DCs. However, addition of anti-IL-10 monoclonal antibodies did not restore the capacity to produce IL-12p70 by TDCM-conditioned slan/M-DC8^+^ cells, suggesting that other downregulatory mechanisms are likely involved.

Another novel finding of this study is the identification of a remarkable plasticity exhibited by blood slan/M-DC8^+^ cells. In fact, we show that blood slan/M-DC8^+^ cells exquisitely acquire all characteristics/features of *ex vivo* isolated tonsil slan/M-DC8^+^ DCs, including morphology, marker expression and functions when conditioned by TDCM for 5 days. In such regard, we found that TDCMs contain discrete amounts of GM-CSF, but not IL-4, which in concert with other factors might drive slan/M-DC8^+^ cell differentiation within the tonsil microenvironment. By contrast, we found that blood slan/M-DC8^+^ cells display a more macrophage-like phenotype when incubated with IL-34. To our knowledge, these are the first data describing effects of IL-34 on circulating slan/M-DC8^+^ cells, which also express the highest levels of CD115/CSF1R among blood leukocytes. CD115 mRNA is highly restricted to the macrophage lineage [36], whose circulating precursors, at least in mice, are the so-called “patrolling” monocytes [37], known to correspond to the “non-classical” CD14^dim^CD16^+^ monocytes in humans [13]. Taken together, all these observations are consistent with the hypothesis of blood slan/M-DC8^+^ cells as a subset of “non-classical” monocytes [12-13] prone to fully differentiate into a more “DC-like” or “macrophage-like” cells depending on the microenvironment of the colonized tissue. In line with this notion, our data indeed show how blood slan/M-DC8^+^ cells differentiate into DCs upon migration into tonsils, as also suggested by de Baey *et al.* [17], who firstly described a M-DC8^+^ cell population in mucosa-associated lymphoid tissues. More broadly, the vision of slan/M-DC8^+^ cells as a yet not fully differentiated subpopulation of blood CD16^+^ monocytes, whose fate is driven by local stimuli, can reconcile the debate in the literature on the identity of these cells. In fact, although blood slan/M-DC8^+^ cells overlap with CD14^dim^CD16^+^ non-classical monocytes, tonsil slan/M-DC8^+^ DCs look and behave differently from their circulating counterpart, displaying bona fide DC functional properties. Despite the definition of slan/M-DC8^+^ cell ontogeny is beyond the scope of this paper, we speculate for a role of blood slan/M-DC8^+^ cells as a potential reservoir of tonsil DCs and spotlight their plasticity and commitment under specific tissue microenvironment. Future studies should be aimed at establishing whether such slan/M-DC8^+^ cell plasticity could be also exploited for therapeutic manipulation of T cell functions in different disease settings.

## MATERIALS AND METHODS

### Cell isolation and culture

PBMCs were isolated from buffy coats of healthy donors by density centrifugation (Ficoll-Paque; GE Healthcare, Little Chalfont, Buckinghamshire, United Kingdom) under endotoxin-free conditions. Then, slan/M-DC8^+^ cells, CD1c^+^ DCs and CD14^+^ monocytes were purified using specific isolation kits (Miltenyi Biotec, Bergisch Gladbach, Germany), to more than 90 % purity, while CD4^+^ T lymphocytes cells were isolated (> 95 % purity) by the EasySep Human CD4 T Cell Enrichment Kit (StemCell Technologies, Vancouver, Canada) [11]. Tonsil samples were obtained from children affected by recurrent, chronic tonsillitis, thus undergoing surgery via cold steel dissection. Tonsils were immediately processed, minced into small fragments, treated for 15 min at 37° with 0.2 mg ml^−1^ Liberase Blendzyme 2 (Roche, Basel, Switzerland), and then processed by gentleMACS dissociator (Miltenyi Biotec) [11]. Tonsil cell suspensions were washed, filtered through a 40 μm cell strainer and ultimately depleted of T and B lymphocytes by CD3 and CD19 MicroBeads (Miltenyi Biotec), to enrich the DCs. Thereafter, tonsil slan/M-DC8^+^ DCs, CD1c^+^ DCs, CD141^+^ DCs, CD14^+^CD11b^+^ monocytes/macrophages and CD303^+^ pDCs were isolated to more than 90 % purity, by fluorescence activated cell sorting (FACS), using a FACSAria II flow cytometer (Becton Dickinson, Franklin Lakes, NJ). After purification, cells were suspended in standard medium [RPMI 1640 medium supplemented with 10 % low-endotoxin fetal bovine serum (FBS, < 0.5 endotoxin U ml^−1^, Sigma-Aldrich, St. Louis, MO)] and cultured for 24 h with 100 U ml^−1^ IFNγ (R&D Systems, Minneapolis, MN) in combination with either 5 μM R848 (InvivoGen, San Diego, CA) or 100 ng ml^−1^ ultrapure LPS (from *E. coli*, 0111:B4 strain, Alexis Biochemicals, San Diego, CA). Alternatively, cells were cultured for 5 days in either tonsil-derived conditioned medium, 50 ng ml^−1^ GM-CSF plus 20 ng ml^−1^ IL-4 (both from Miltenyi Biotec), or 100 ng ml^−1^ IL-34 (R&D system). For morphological analysis, cells were subjected to cytospin and stained by the May-Grunwald/Giemsa procedure. Pictures were taken using a Leica DFC 300FX Digital Color Camera on a Leica DM 6000 B microscope. All experimental procedures were approved by the institutional review boards of the University of Verona and Spedali Civili of Brescia. Retrospective analysis of archival material (see below) was conducted in compliance with the Declaration of Helsinki and with policies approved by the Ethics Board of Spedali Civili di Brescia. Human samples were obtained following informed written consent.

### Immunohistochemistry

Tissue blocks containing formalin-fixed paraffin-embedded (FFPE) tonsils were retrieved from the tissue bank of the Department of Pathology (Spedali Civili di Brescia, Brescia, Italy). Four-micron thick tissue sections were used for immunohistochemical staining. slan/M-DC8^+^ cells were specifically identified by using primary antibodies towards the 6-sulfo LacNAc residue (slan/M-DC8) on PSGL-1, namely clone DD1, as previously reported [10]. Other antigens were identified using antibodies listed in Supplementary Table S1. The primary immune reaction was revealed using Novolink Polymer (Leica Microsystems, Wetzlar, Germany) followed by 3, 3′-diaminobenzidine (DAB). For double immunohistochemistry, after completing the first immune reaction, the second one was visualized using Mach 4 MR-Alkaline Phosphatase (AP) (Biocare Medical), followed by Ferangi Blue (Biocare Medical, Concord, CA) as chromogen. For triple immunohistochemistry, after completing the second immune reaction, sections were incubated with primary antibodies to Ki-67 and revealed using a biotinylated system followed by streptavidinconjugated with AP (Dako, Glostrup, Denmark) with New Fucsin as chromogen.

### Generation of tonsil derived conditioned medium (TDCM) and TDCM-conditioned cells

TDCM was generated by culturing tonsil cell suspension (10*10^6^ ml^−1^, *n* = 8) in RPMI plus 10 % FBS for 24 h. Cell-free supernatants were then collected and stored at - 20° C. Each TDCM was diluted 1:5 in RPMI plus 10 % FBS immediately before its addition to blood slan/M-DC8^+^ cells, CD1c^+^ DCs or CD14^+^ monocytes for subsequent incubation. After 5 d, cells were harvested, extensively washed and used for different functional assays. In selected experiments, anti-IL-10 mAbs, or their IgG2a isotype controls (10 μg ml^−1^, both from R&D system), were added to slan/M-DC8^+^ cells during the 5 d-incubation with TDCM, as well as during the subsequent 24 h-activation with IFNγ plus LPS.

### Flow cytometry analysis

For phenotypic studies, typically 2.5×10^5^ PBMCs, 5×10^5^ cells from tonsil cell suspensions or 10^4^ *in vitro* stimulated slan/M-DC8^+^ cells were initially incubated for 10 min in 50 μl Phosphate Buffer Solution (PBS) containing 5 % human serum (to prevent nonspecific binding), and then stained for 15 min at room T using the monoclonal antibodies listed in Supplementary Table S2. Sample fluorescence was measured by an eight-color MACSQuant Analyzer (Miltenyi Biotec), while data analysis was performed by FlowJo software Version 8.8.7 (Tree Star Inc., Stanford, CA) [11]. Cell viability was analyzed using Vybrant® DyeCycle™ Violet (Life Technologies, Carlsbad, CA) [11]. Phenotypic analysis under the various experimental conditions was performed on live cells, identified as Vybrant-negative cells (in the case of TDCM-conditioned/stimulated slan/M-DC8^+^ cells) or PI-negative cells (in the case of tonsil cell suspensions) [11]. The mean fluorescence intensity (MFI) relative to each molecule was obtained by subtracting either the MFI of the correspondent isotype control, or cell autofluorescence (fmo).

### T cell proliferation assays

For allogeneic assays, 1.25-5×10^3^ slan/M-DC8^+^ DCs, CD1c^+^ DCs, CD141^+^ DCs, CD14^+^CD11b^+^ monocytes/macrophages and CD303^+^ pDCs, sorted from tonsils, were co-cultured with 5×10^4^ CFSE-labeled allogeneic CD4^+^ T lymphocytes in U-bottom 96-well plates [11]. For autologous assays, 5×10^3^ freshly isolated or 5-d TDCM-conditioned slan/M-DC8^+^ cells, CD1c^+^ DCs and CD14^+^ monocytes were co-cultured with 5×10^4^ CFSE-labeled autologous CD4^+^ T lymphocytes in U-bottomed 96-well plates, in the absence or presence of 5 μg ml^−1^ tetanus toxoid (TT) [11]. For both allogeneic and autologous assays, T-cell proliferation was assessed after 7 days by measuring CFSE dilution by flow cytometry [11].

### Analysis of cytokine production

Total cell suspensions from tonsils were analyzed for TNFα and IL-12p70 production by specific cytokine secretion assays (Miltenyi Biotec) [11]. Briefly, 5×10^5^ tonsil cells were incubated with 100 U ml^−1^ IFNγ in combination with either 100 ng ml^−1^ ultrapure LPS or 5 μM R848 in standard medium at 37°C either for 4 h, to optimally detect TNFα secretion, or for 12 h, after a 6 h pre-incubation in standard medium, to optimally detect IL-12p70 secretion. Percentages of cytokine secreting cells were then identified as cytokine-positive cells among total slan/M-DC8^+^ DCs, CD1c^+^ DCs, CD14^+^CD11b^+^ monocytes/macrophages and CD303^+^ pDCs, gated as shown in detail in Supplementary Figure S1. TNFα and IL-12p70 levels present in cell-free supernatants harvested from either blood or TDCM-conditioned slan/M-DC8^+^ cells, and stimulated as detailed in legend to Figure 6, were measured by specific ELISA kits from eBioScience (San Diego, CA; sensitivity: 4 pg ml^−1^). The levels of IL-10, GM-CSF and IL-4 in TDCMs were measured by ELISA kits, purchased from eBioScience, BioLegend (San Diego, CA) and Mabtech (Cincinnati, OH), respectively. Detection limits of these ELISA were: 2 pg ml^−1^ for IL-10, 3 pg ml^−1^ for GM-CSF and IL-4

### Statistical analysis

Data are expressed as means ± SEM of the number of experiments indicated in each Figure legend. Statistical analysis, including one-way or two-way analysis of variance followed by Bonferroni's post hoc test, was performed by Prism Version 5.0 software (GraphPad Software, Inc., La Jolla, CA).

## SUPPLEMENTARY TABLES AND FIGURES


