# Correction: slan/M-DC8+ cells constitute a distinct subset of dendritic cells in human tonsils

**DOI:** 10.18632/oncotarget.12418

**Published:** 2016-10-03

**Authors:** Alessandra Micheletti, Giulia Finotti, Federica Calzetti, Silvia Lonardi, Elisa Zoratti, Mattia Bugatti, Stefania Stefini, William Vermi, Marco A. Cassatella

Present Title: slan/M-DC8+ cells constitute a distinct subset of dendritic cells in human tonsils

Present Keywords: slan/M-DC8+ cells, dendritic cells, monocytes, tonsil, differentiation, Immunology and Microbiology Section, Immune response, Immunity

Correct Title: slanDCs/M-DC8+ cells constitute a distinct subset of dendritic cells in human tonsils

Correct Keywords: slanDCs/M-DC8+ cells, dendritic cells, monocytes, tonsil, differentiation, Immunology and Microbiology Section, Immune response, Immunity

Authors sincerely apologize for this oversight.

Original article: Oncotarget. 2016; 7(1):161-175. doi: 10.18632/oncotarget.6660.

