# Supplementary material for: slan/M-DC8+ cells constitute a distinct subset of dendritic cells in human tonsils
Source: Oncotarget. 2015 Dec 18;7(1):161–75. doi: 10.18632/oncotarget.6660 (PMC4807990; doi:10.18632/oncotarget.6660)
Supplement: Supplementary file 1 [file oncotarget-07-0161-s001.pdf]

slan/M-DC8$^+$ cells constitute a distinct subset of dendritic cells in human tonsil

Supplementary Table S1. List of the antibodies used for immunohistochemistry studies.

Reagent	Clone	Dilution	Isotype	Source
**BCL6**	IG191E/A8	1:300	mIgG1	kindly provided by G. Roncador (Centro Nacional de Investigaciones Oncológicas Madrid, Spain)
**CD1a**	010	1:50	mIgG1	Dako
**CD3**	SP7	1:100	rabbit	Thermo Scientific, Waltham, MA
**CD4**	4B12	1:40	mIgG1	Thermo Scientific
**CD8**	C8/144B	1:30	mIgG1	Dako
**CD11b**		1:300	rabbit polyclonal	Sigma-Aldrich
**CD14**	7	1:50	mIgG2a	Novocastra Laboratories, Newcastle upon Tyne, United Kingdom
**CD66b**	G10F5	1:200	mIgM	BioLegend, San Diego, CA
**CD83**	1H4b	1:150	mIgG1	Novocastra Laboratories
**DD1**	DD1	1:60	mIgM	kindly provided by Knut Schäkel (University Hospital Heidelberg, Heidelberg, Germany)
**Keratin (wide spectrum-CKP)**	MNF116	1:100	mIgG1	Dako
**Ki-67**	MM1	1:100	mIgG1	Novocastra Laboratories

Supplementary Table S2. List of the antibodies used for flow cytometry.

Antibody	Clone	Isotype	Source
**AlexaFluor488 anti-human CD1c**	L161	mIgG1	BioLegend
**AlexaFluor647 anti-human CX3CR1**	2A9-1	rat IgG2b	BioLegend
AlexaFluor647 rat IgG2b	RTK4530		BioLegend
**APC anti-human CD11b**	ICRF44	mIgG1	BioLegend
**APC anti-human CD11c**	MJ4-27G12	mIgG2b	Miltenyi Biotec
**APC anti-human CD14**	TUK4	mIgG2a	Miltenyi Biotec
**APC anti-human CD141 (BDCA-3)**	AD5-14H12	mIgG1	Miltenyi Biotec
**APC-Cy7 anti-human HLA-DR**	L243	mIgG2a	BioLegend
**Brilliant Violet 510 anti-human CD45**	HI30	mIgG1	BioLegend
**FITC anti-human CD14**	TÜK4	mIgG2a	Miltenyi Biotec
**FITC anti-human CD141**	AD5-14H12	mIgG1	Miltenyi Biotec
**FITC anti-human CD303**	AC144	mIgG1	Miltenyi Biotec
**FITC anti-human Slan (M-DC8)**	DD1	mIgM	Miltenyi Biotec
**PE anti-human CD1c (BDCA-1)**	AD5-8E7	mIgG2a	Miltenyi Biotec
**PE anti-human CD11b**	ICRF44	mIgG1	BioLegend
**PE anti-human CD115**	9-4D2-1E4	rat IgG1	BioLegend
**PE anti-human CD14**	TUK4	mIgG2a	Miltenyi Biotec
**PE anti-human CD16**	3G8	mIgG1	BioLegend
**PE anti-human CD163**	GHI/61	mIgG1	BioLegend
**PE anti-human CD40**	HB14	mIgG1	BioLegend
**PE anti-human CD80**	2D10	mIgG1	BioLegend
**PE anti-human CD83**	HB15	mIgG1	Miltenyi Biotec
**PE anti-human CD86**	IT2.2	mIgG2b	BioLegend
**PE anti-human FcεRI**	CRA1	mIgG2b	Miltenyi Biotec
**PE anti-human CD206**	15-2	mIgG1	BioLegend
**PE anti-human CD209 (DC-SIGN)**	9E9A8	mIgG2a	BioLegend
PE mouse IgG1	MOPC-21	mIgG1	BioLegend
**PE-Cy7 anti-human CD19**	HIB19	mIgG1	BioLegend
**PE-Cy7 anti-human CD3**	UCHT1	mIgG1	BioLegend
**PerCP-Cy5.5 anti-human CD16**	3G8	mIgG1	BioLegend
**Vioblue anti-human CD11c**	MJ4-27G12	mIgG2b	Miltenyi Biotec

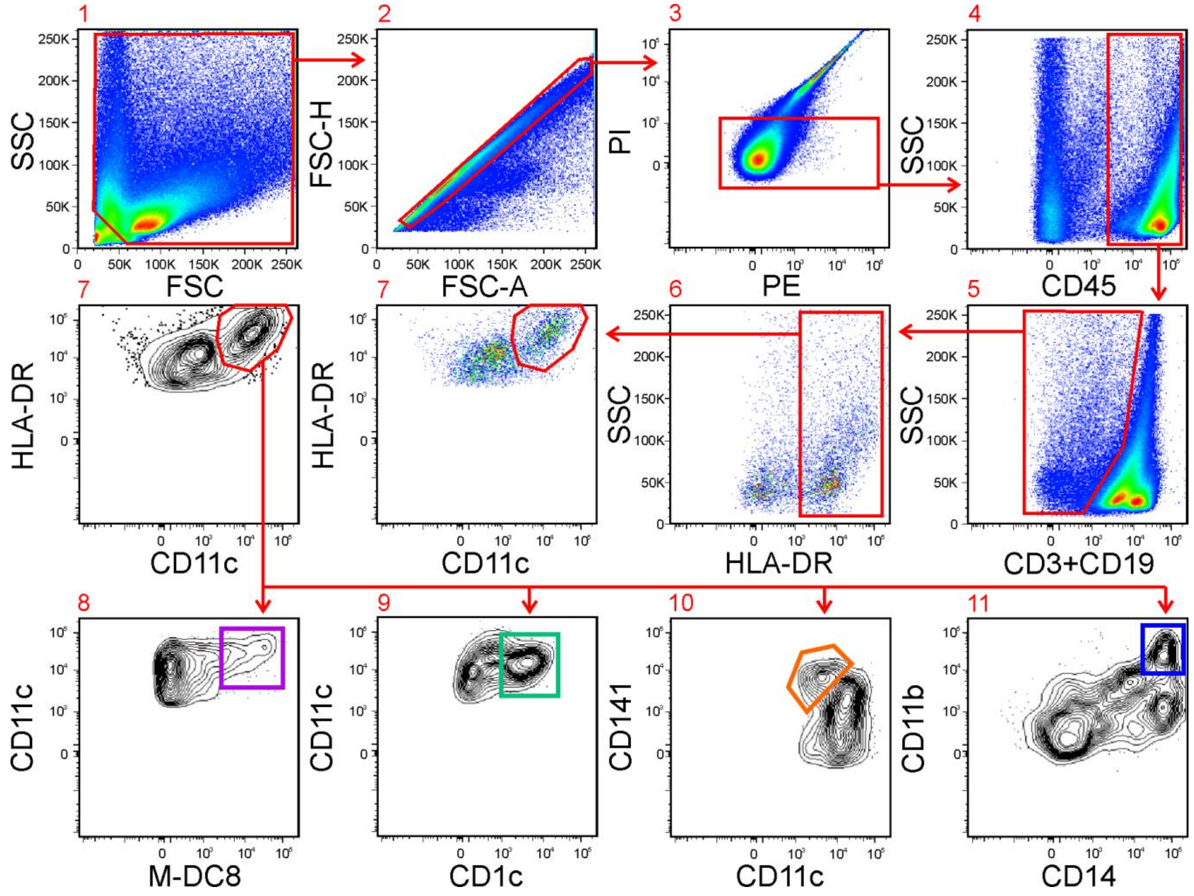

Supplementary Figure S1. Gating strategy to distinctively identify slan/M-DC8$^+$ DCs, CD1c$^+$ DCs, CD141$^+$ DCs and CD14$^+$CD11b$^+$ monocytes/macrophages in human tonsils.

Single cell suspensions from tonsils were processed for flow cytometry analysis to identify slan/M-DC8$^+$ DCs, CD1c$^+$ DCs, CD141$^+$ DCs and CD14$^+$CD11b$^+$ monocytes/macrophages. Steps 1-4 were sequentially used to exclude cell debris (1), doublets (2), dead cells (3), and, ultimately, to gate CD45$^+$ leukocytes (4). Subsequently, in steps 5-7, analysis was performed on CD3/CD19-negative cells (5). Within the latter cells, HLA-DR-positive (6) and subsequently HLA-DR$^+$CD11c$^+$ cell populations (7) were gated. The latter HLA-DR$^+$CD11c$^+$ population includes, in fact, all myeloid DCs, macrophages and monocytes. Steps 8-11 show the specific combination of markers used to gate each myeloid population type: slan/M-DC8$^+$ DCs (purple gate, 8), CD1c$^+$ DCs (green gate, 9), CD141$^+$ DCs (orange gate, 10) and CD14$^+$/CD11b$^+$ monocytes/macrophages (blue gate, 11).

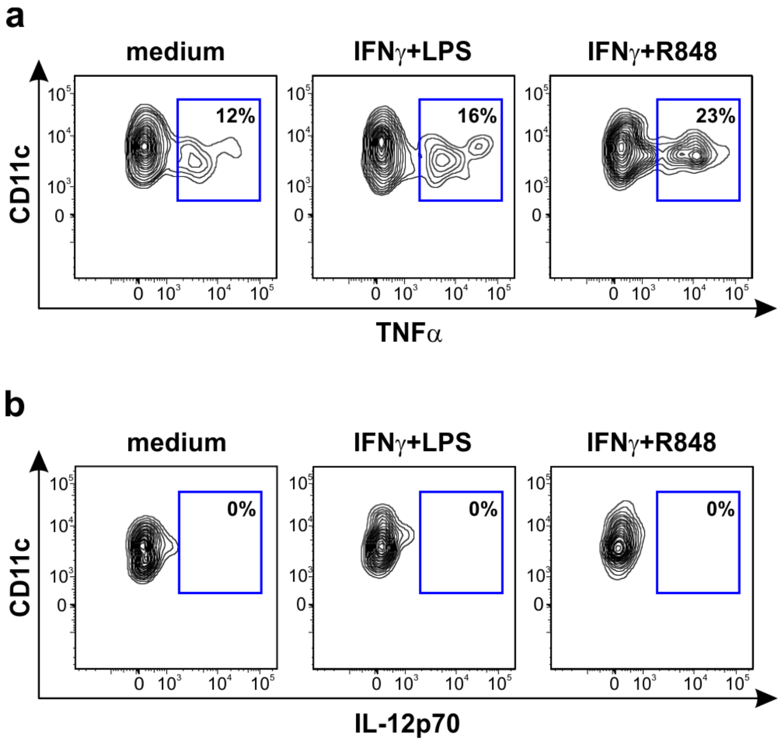

Supplementary Figure S2. TNFα and IL-12p70 secretion by tonsil slan/M-DC8^{+} DCs.

Tonsil cell suspensions were incubated with or without 100 U ml^{-1} IFNγ plus either 100 ng ml^{-1} LPS or 5 μM R848, either for 4 h (to detect TNFα secretion), or for 12 h, after a 6 h pre-incubation (to detect IL-12p70 secretion). Contour plots display a representative experiment illustrating the percentage of TNFα- (a) or IL-12p70- (b) secreting tonsil slan/M-DC8^{+} DCs.
